# Diagnostic and prognostic role of electrocardiogram in acute myocarditis: A comprehensive review

**DOI:** 10.1111/anec.12726

**Published:** 2019-11-28

**Authors:** Carmelo Buttà, Luca Zappia, Giulia Laterra, Marco Roberto

**Affiliations:** ^1^ Cardiology Unit Department of Clinical and Experimental Medicine University of Messina Messina Italy; ^2^ Department of Cardiology Cardiocentro Ticino Lugano Switzerland

**Keywords:** atrial fibrillation/atrial arrhythmias, cardiac anatomy, electrocardiography, MRI, ventricular tachycardia/fibrillation

## Abstract

**Background:**

Acute myocarditis represents a challenging diagnosis as there is no pathognomonic clinical presentation. In patients with myocarditis, electrocardiogram (ECG) can display a variety of non‐specific abnormalities. Nevertheless, ECG is widely used as an initial screening tool for myocarditis.

**Methods:**

We researched all possible ECG alterations during acute myocarditis evaluating prevalence, physiopathology, correlation with clinical presentation patterns, role in differential diagnosis, and prognostic yield.

**Results:**

The most common ECG abnormality in myocarditis is sinus tachycardia associated with nonspecific ST/T‐wave changes. The presence of PR segment depression both in precordial and limb leads, a PR segment depression in leads with ST segment elevation, a PR segment elevation in aVR lead or a ST elevation with pericarditis pattern favor generally diagnosis of perimyocarditis rather than myocardial infarction. In patients with acute myocarditis, features associated with a poorer prognosis are: pathological Q wave, wide QRS complex, QRS/T angle ≥ 100°, prolonged QT interval, high‐degree atrioventricular block and malignant ventricular tachyarrhythmia. On the contrary, ST elevation with a typical early repolarization pattern is associated with a better prognosis.

**Conclusions:**

ECG alterations in acute myocarditis could be very useful in clinical practice for a patient‐tailored approach in order to decide appropriate therapy, length of hospitalization, and frequency of followup.

## 1 INTRODUCTION

Myocarditis represents an inflammatory disease characterized by inflammatory infiltrates within the myocardium associated with myocyte degeneration and necrosis of nonischemic origin (Caforio et al., 2013). Diagnosis may be challenging because there is no pathognomonic clinical presentation and the disease may mimic a variety of noninflammatory myocardial diseases (Caforio, Marcolongo, Basso, & Iliceto, 2015; Caforio et al., 2013). Particularly, it may be difficult to differentiate between pericarditis, myocarditis, myopericarditis, and perimyocarditis; this diagnostic challenge may be reflected in different prevalence or incidence of electrocardiogram (ECG) changes in various study populations.

Clinical presentation of myocarditis is heterogeneous, and possible clinical pictures include (a) asymptomatic course, (b) presentation with chest pain mimicking myocardial infarction (MI), also known as acute coronary syndrome (ACS)‐like myocarditis, (c) presentation with symptoms and signs of heart failure (HF) typically with impaired systolic function, and (d) life‐threatening presentation with cardiogenic shock and/or malignant arrhythmias, also known as fulminant myocarditis (FM) (Biesbroek, Beek, Germans, Niessen, & van Rossum, 2015; Caforio et al., 2015; Kindermann et al., 2012). FM on presentation is more common in giant cell myocarditis (GCM) and eosinophilic myocarditis (EM) as compared with myocarditis of other origin (Granér, Lommi, Kupari, Räisänen‐Sokolowski, & Toivonen, 2007; Kuchynka et al., 2016).

Imaging methods such as cardiac magnetic resonance imaging (MRI) are useful tools to diagnose and monitor the progression of disease, but the gold standard for diagnosis remains endomyocardial biopsy (Caforio et al., 2013). 2013 ESC Task Force proposed criteria for selection of patients with suspected myocarditis for whom endomyocardial biopsy should be considered and ECG was identified as a useful tool in this context (Caforio et al., 2013).

In patients with myocarditis, ECG can display a variety of abnormalities, none of which pathognomonic (Punja et al., 2010). Sensitivity of ECG for diagnosis of myocarditis is low and it is estimated at 47%, but specificity remains unknown (Cooper, 2009; Dennert, Crijns, & Heymans, 2008). Nevertheless, ECG is widely used as an initial screening tool for myocarditis (Fung, Luo, Qiu, Yang, & McManus, 2016).

The purpose of this article was to provide a comprehensive review of all possible ECG alterations during acute myocarditis evaluating prevalence, physiopathology, correlation with clinical presentation patterns, role in differential diagnosis and prognostic yield. We will subdivide this article into the following sections: PR segment, QRS complex, ST segment, T wave, QT interval, and arrhythmias.

## 2 PR SEGMENT

PR segment depression is a possible but uncommon ECG finding in pure acute myocarditis, being globally more frequent in myopericarditis and pericarditis (Dennert et al., 2008; Eichhorn et al., 2019), where it represents the first of 4 characteristic ECG evolution stages (Chan, Brady, & Pollack, 1999; Punja et al., 2010). Although the pericardial sac itself has no electrical activity, inflammation of the pericardium can disrupt the action potential in the epicardium (de Bliek, 2018); as a result, the involvement of the atria is responsible for depression of the PR segment that expresses atrial repolarization irregularities. Primitive inflammation of atrial myocardium, as observed in pure myocarditis not secondary to pericarditis, can cause similar alterations.

**Figure 1:**
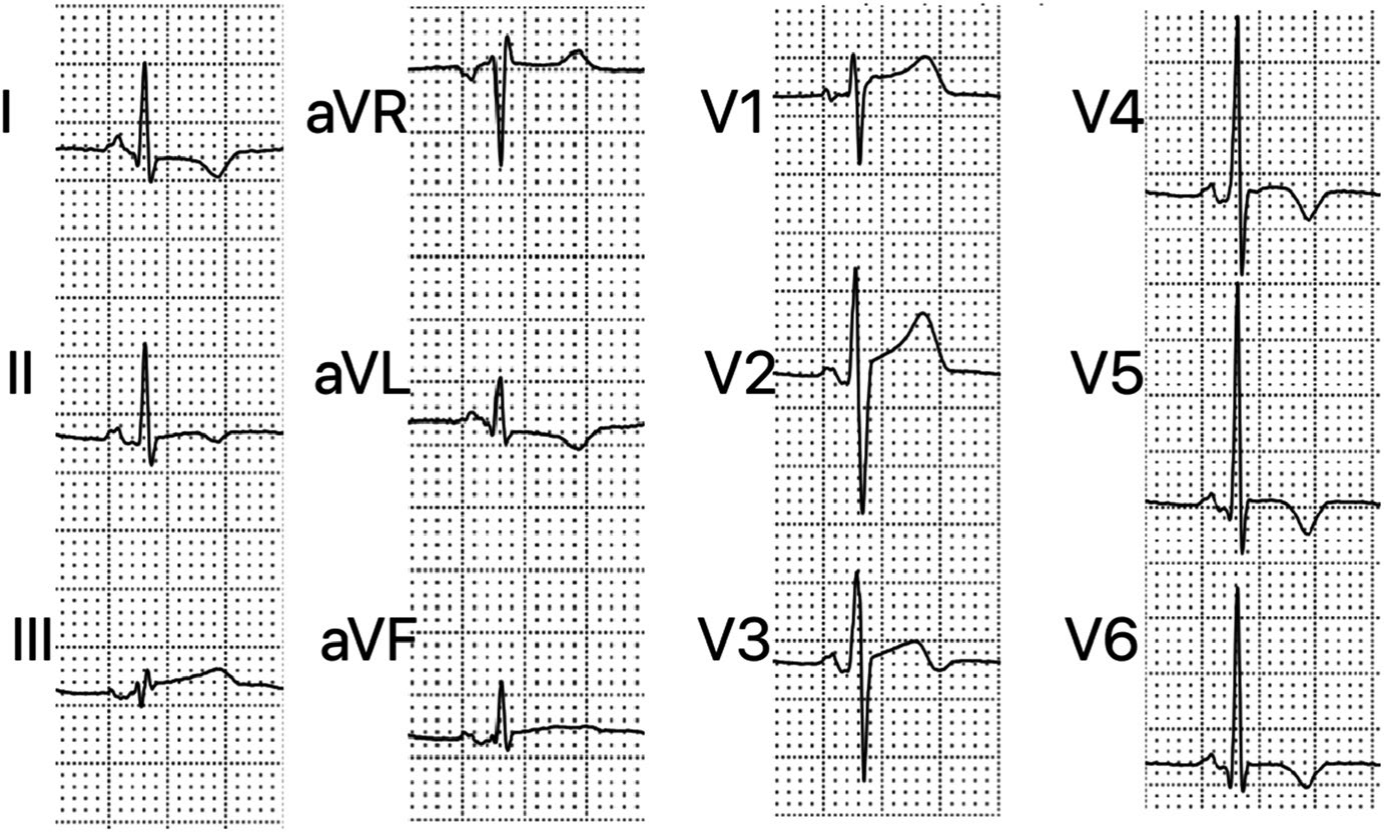
Electrocardiogram performed 11 days after symptom onset in a 19‐year‐old patient with perimyocarditis. It shows the following: PR segment depression in inferior and V2‐V6 leads, PR segment elevation in aVR, ST elevation in inferior and V1‐V3 leads, and T‐wave inversion in I, II, aVL, and V3‐V6 leads

In clinical studies on pericarditis/myocarditis, PR depression was often defined only qualitatively; however, when a quantitative analysis was performed, a cutoff ≥0.5 mm was used to define a pathological PR depression (Porela, Kytö, Nikus, Eskola, & Airaksinen, 2012). It generally occurs diffusely in limb and precordial leads, with the exception of aVR and V1, which may have reciprocal PR segment elevation (see Figure 1). PR abnormalities are seen most readily in leads II, aVR, aVF, and V4‐V6 and tend to be more prominent in leads II, V5, and V6 (Chan et al., 1999; Demangone, 2006). PR segment depression may be seen also in patients with atrial MI or early repolarization extended to the atria (de Bliek, 2018; Liu, Greenspan, & Piccirillo, 1961). However, there is no established consensus for diagnosis of atrial MI, and PR segment displacement in this setting is not yet validated in prospective studies (Yıldız et al., 2018). Nevertheless, the presence of PR segment depression both in precordial and limb leads, a PR segment depression in leads with ST segment elevation or a PR segment elevation in aVR lead favor generally diagnosis of perimyocarditis rather than MI (Pollak & Brady, 2012; Porela et al., 2012).

## 3 QRS COMPLEX

As underlined by 2013 ESC Task Force, QRS complex alterations in acute myocarditis include low voltages, abnormal Q waves, and intraventricular conduction delay/bundle branch block (BBB) (Caforio et al., 2013).

Widespread low QRS voltage is a possible ECG manifestation of myopericarditis with pericardial effusion as a result of increased resistance from the accumulated fluid (Chan et al., 1999; Punja et al., 2010). However, in a study by Nakashima H et al, 18% of patients with acute myocarditis had a significant decrease in QRS amplitude in limb and chest leads during the acute phase of illness compared with QRS voltage recorded before illness and during the convalescence stage regardless of pericardial effusion (Nakashima, Honda, & Katayama, 1994). Proposed mechanisms of low QRS voltages in pure myocarditis (particularly FM) include edema of the ventricular wall, pulmonary edema, and peripheral edema (Lee et al., 2013; Madias, 2007).

The two most common QRS alterations in acute myocarditis are the presence of abnormal Q waves and intraventricular conduction delay/BBB. Pathological Q waves are typical of acute MI, but they can be present in myocarditis too with the same characters (Chan et al., 1999; Thygesen et al., 2018). They are one of the possible ECG manifestations of ACS‐like myocarditis, but they are also described in patients with FM (Demangone, 2006; Punja et al., 2010). Previous reports showed a prevalence of abnormal Q waves in acute myocarditis generally lower than 20%, but an old study by Nakashima et al reported prevalence as high as 68% (Dec et al., 1992; Nakashima et al., 1994). The broad variability among published studies in prevalence of abnormal Q waves and other ECG alterations in myocarditis could be at least in part explained by (a) their transient nature (Di Bella et al., 2012; Nakashima et al., 1994); (b) progressive changes in diagnostic techniques with widespread diffusion of cardiac MRI and high‐sensitivity troponins allowing identification of patients with less evident clinical presentation and, as a result, their inclusion in more recent clinical studies. Regarding ECG localization of Q waves, some studies showed that Q waves were more frequently distributed in inferior and lateral leads; moreover, a study by Deluigi et al showed that patients with inferolateral Q waves had a lateral distribution of late gadolinium enhancement (LGE), predominantly transmural, at MRI (Deluigi et al., 2013; Jhamnani, Fuisz, & Lindsay, 2014; Nakashima et al., 1994). In myocarditis, the early disappearance of Q waves suggests a reversible myocardial lesion and the resolution of the inflammatory process (Nakashima et al., 1994). Nevertheless, the presence of pathologic Q waves has been associated with a poorer prognosis in FM, especially when associated with ST segment elevation (Elamm, Fairweather, & Cooper, 2012; Ginsberg & Parrillo, 2013; Morgera et al., 1992; Take, Sekiguchi, Hiroe, & Hirosawa, 1982).

Intraventricular conduction delay and BBB are alterations secondary to organic damage of myocardiocytes or electrical conduction system. Generally, the prevalence of wide QRS complex in myocarditis is variable and dependent on the severity of clinical presentation. In an old study by Nakashima et al, BBB occurred in 55% of patients with an equal prevalence of right and left BBB (Nakashima et al., 1994). Another study by Sawamura A et al showed a wide QRS in 66% of patients with FM supported by percutaneous venoarterial extracorporeal membrane oxygenation and, among these, in 80% of nonsurvivor patients (Sawamura et al., 2018). Finally, myocardial involvement in Chagas disease is associated with a high prevalence of right BBB and left anterior fascicular block (Rojas et al., 2018).

The presence of wide QRS complex has been associated with a poorer prognosis, particularly in FM, where it can be one of the earliest clinical signs (Elamm et al., 2012; Ginsberg & Parrillo, 2013; Ukena et al., 2011; Wang et al., 2019). A study by Morgera B et al showed that abnormal QRS complexes were associated with more severe left ventricular impairment and with a higher frequency of hypertrophy and fibrosis (according to histologic examination) (Morgera et al., 1992). Another study by Jhamnani S et al showed that, in acute myocarditis, the presence of an abnormal QRS complex was related to a higher prevalence of left ventricular ejection fraction below 45% (Jhamnani et al., 2014).

Finally, QRS fragmentation, a well‐known ECG marker of myocardial scar/fibrosis (Pietrasik & Zaręba, 2012), defined as the presence of an additional R wave in at least two contiguous leads, has been recently shown to have a grossly topographic correlation with areas of distribution of LGE at cardiac MRI in a preliminary study on myocarditis (Ferrero et al., 2019).

## 4 ST SEGMENT

**Figure 2:**
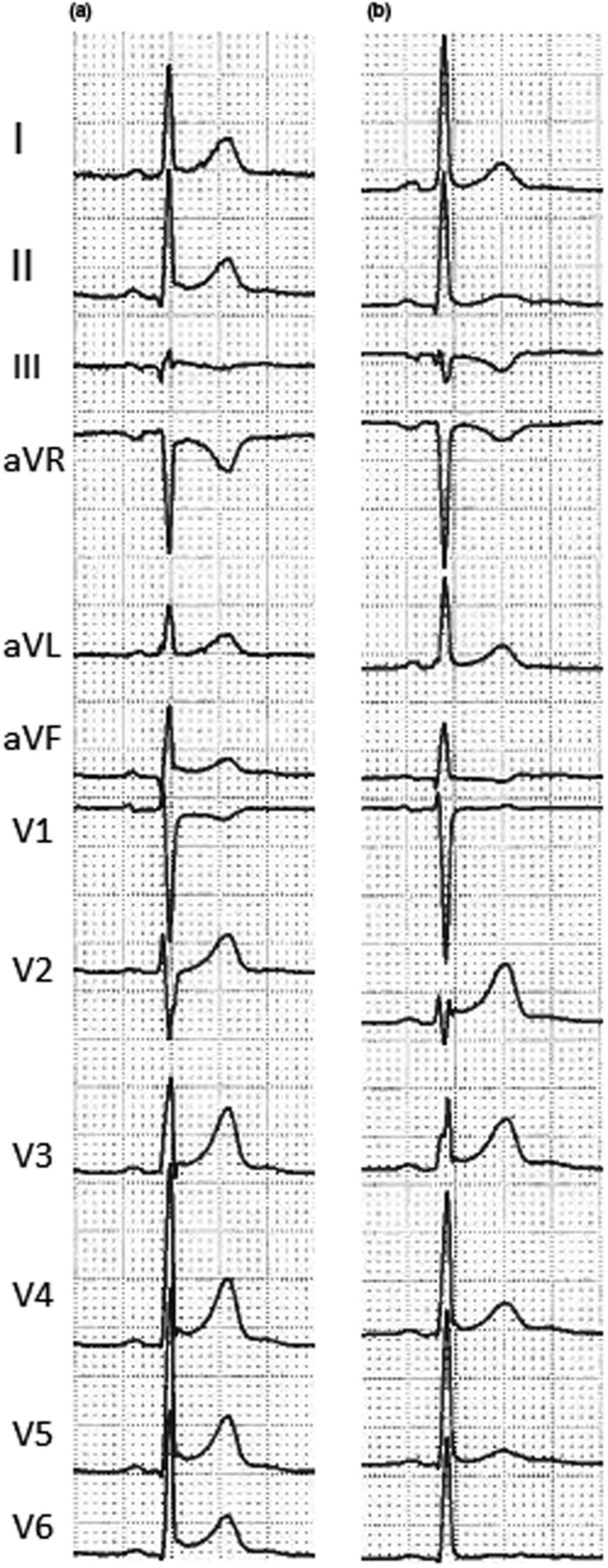
Electrocardiogram performed at admission (a) and 3 days later (b) in a 30‐year‐old patient with perimyocarditis. Comparison between these two ECGs shows the following: disappearance of ST elevation with pericarditis pattern in inferior and lateral leads, disappearance of ST depression in aVR lead, and onset of T‐wave inversion in III e aVF leads

ST segment elevation is the most common ST change in acute myocarditis although ST depression is also possible. ST elevation in subepicardial lesion is due to the current of injury during phase 4 of action potential that causes a displacement of the isoelectric line downward; consequently, during phase 2 of action potential, ST segment appears upward shifted (de Bliek, 2018; Oreto, 2008). This abnormality is generally more evident in perimyocarditis (Demangone, 2006; Pollak & Brady, 2012). However, ST elevation may be found in any clinical presentation pattern of pure acute myocarditis. Dec and colleagues found that, in patients with biopsy‐proven ACS‐like myocarditis, ST elevation was the most common ischemic‐type ECG abnormality (Dec et al., 1992). Prevalence of ST elevation in acute myocarditis ranges from 24% to 73% among various studies (Angelini et al., 2000; Dec et al., 1992; Deluigi et al., 2013; Di Bella et al., 2012; Nakashima et al., 1994). This high variability is at least in part due to the variable time interval comprised between symptom onset and patient presentation to the hospital. Indeed, as for other ECG alterations in myocarditis, ST elevation is usually transient and it could disappear within 24 and 48 hr, respectively, in 49% and 74% of all patients displaying ST elevation at presentation (see Figure 2) (Di Bella et al., 2012; Nucifora et al., 2013).

**Figure 3:**
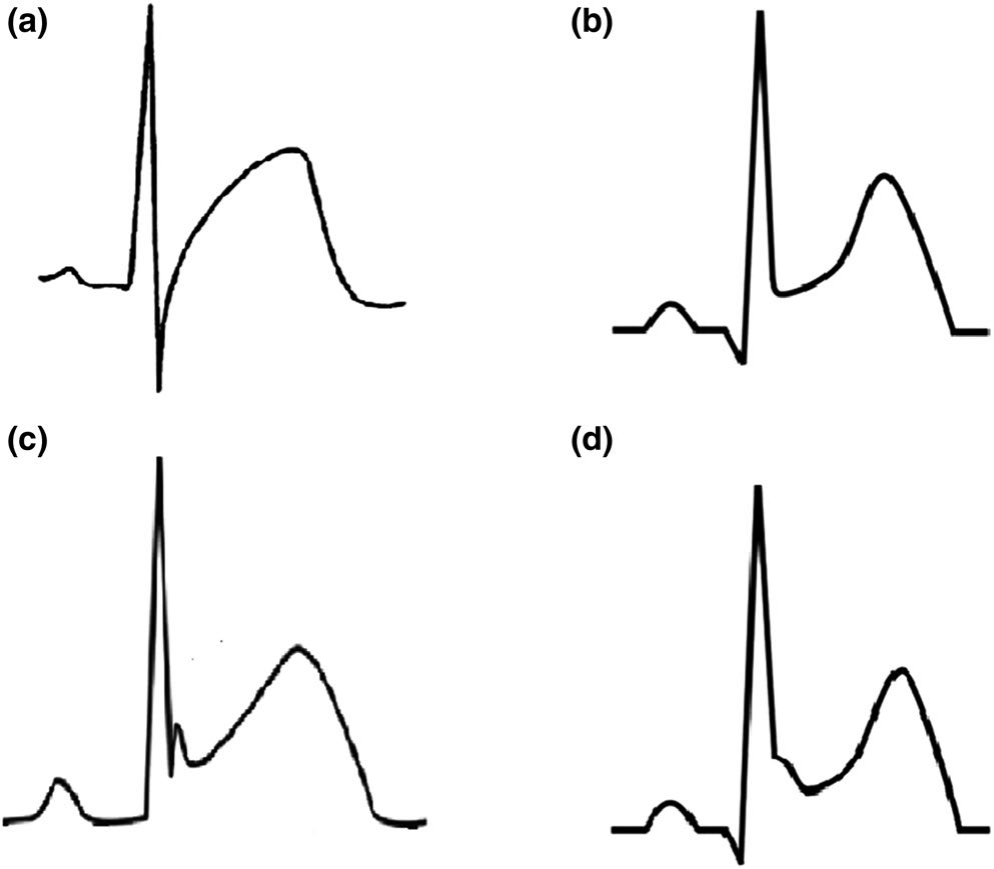
Possible ST‐elevation patterns in acute myocarditis: acute coronary syndrome‐like pattern (a), pericarditis pattern without terminal QRS notching or slurring (b), pericarditis pattern with terminal QRS notching (c), and pericarditis pattern with terminal QRS slurring (d)

**Figure 4:**
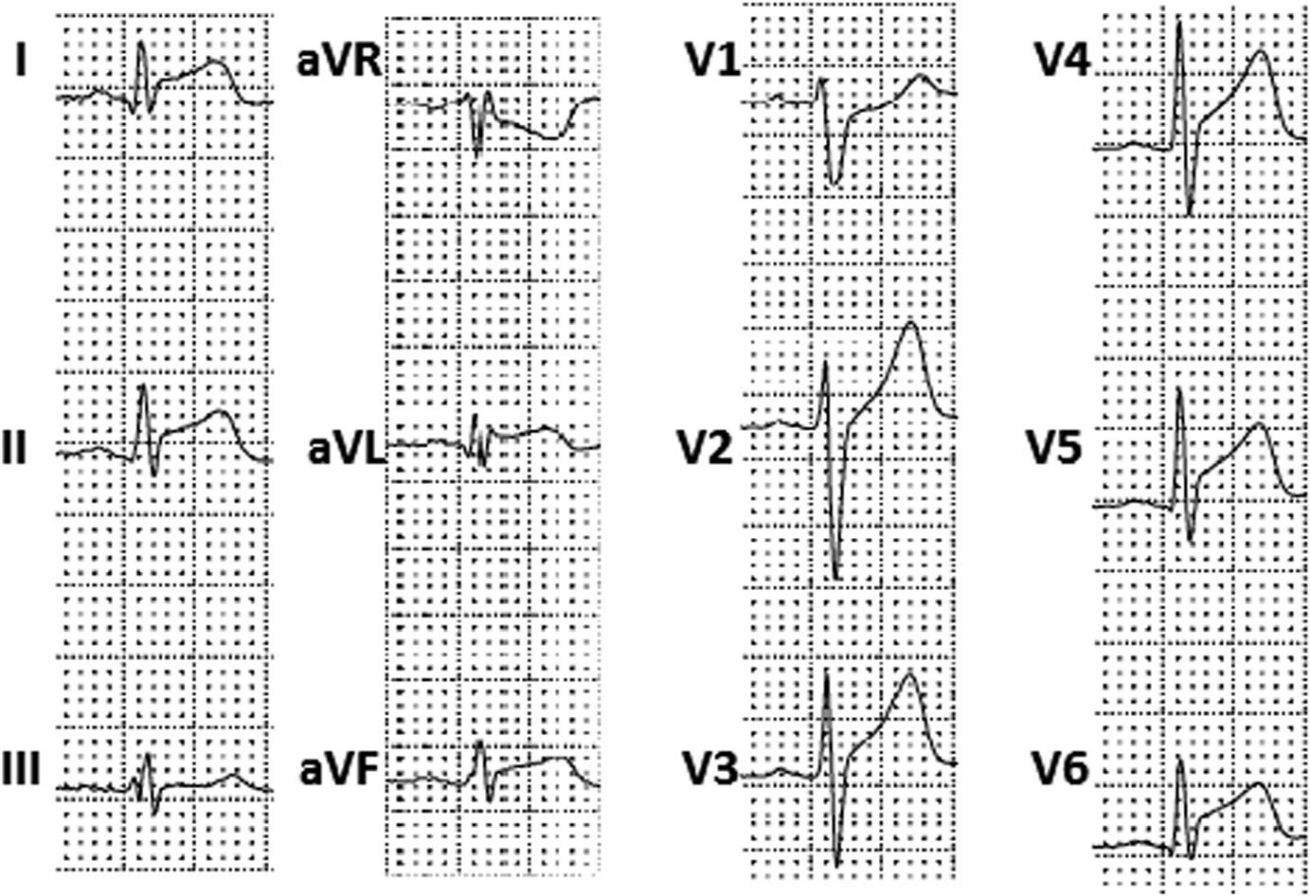
Electrocardiogram performed 1 day after hospital admission in a 18‐year‐old patient with pure acute myocarditis. It shows a diffuse ST elevation with pericarditis pattern without terminal QRS notching or slurring and a reciprocal ST depression in aVR and V1 leads

In myocarditis, two ST‐elevation patterns have been described: a pericarditis pattern or a typical MI‐like pattern (see Figure 3) (Hanna & Glancy, 2015). Pericarditis pattern is more common in acute perimyocarditis and it is characterized by a J‐point elevation and an upward concave shape of ST segment, with or without terminal QRS notching or slurring (see Figures 1, 2, 3, 4). The elevation is usually less than 5 mm and involves diffusely both limb and precordial leads (I, II, III, aVF, aVL, V2‐V6) with the exception of aVR and V1 (which often present reciprocal ST depression) (see Figure 4) (Chan et al., 1999). This pattern may be confused with the benign ST diffuse elevation in early repolarization pattern that may be associated with a PR segment depression in the inferolateral leads too (Birnbaum, Perez Riera, & Nikus, 2019). In these cases, the global clinical picture or the comparison with a previous ECG may be useful for differential diagnosis. On the contrary, typical MI‐like pattern is characterized by a J‐point elevation and an upsloping flat or convex ST segment in at least two contiguous leads generally without reciprocal ST depression. A clear territorial distribution on ECG is not always visible. No strict correlation was found between location of ST elevation on surface ECG and areas of distribution of necrosis/scar as assessed by MRI in LGE sequences (Deluigi et al., 2013; Di Bella et al., 2012; Nucifora et al., 2013). A study by Karjalainen et al showed that, in young men with ACS‐like myocarditis, patients with larger extension of myocardial damage (as assessed by peak creatine kinase MB value) had higher maximum ST elevation in a single lead (Karjalainen & Heikkilä, 1986). In another study by Nucifora G et al, patients with larger areas of LGE had a higher sum ST elevation of all leads and a higher rate of late ST normalization (*>*24 hr) (Nucifora et al., 2013). Taken together, these data suggest that ST elevation is of limited utility for the assessment of the location of areas of necrosis/scar but could be very useful for a fast bedside estimation of the extent of myocardial damage in patients with acute myocarditis (Nucifora et al., 2013). However, recently, a study by Oka E et al showed that ST elevation with a typical early repolarization pattern, defined as terminal QRS notching or slurring with an amplitude of >0.1 mV in at least two inferior or lateral leads, is associated with a better prognosis (Oka et al., 2019).

ST elevation represents a diagnostic dilemma for physicians because of the widespread differential diagnosis including ACS. Clinical factors supporting a diagnosis of myocarditis include lower patient age (especially if below 40 years), complaint of recent viral illness, slowly evolving ECG changes involving more than one vascular territory, and diffuse or absent (rather than focal) wall motion abnormalities on echocardiogram (Punja et al., 2010). ST elevation in multiple coronary distribution territories, especially when seen in patients who are clinically stable, favors inflammation over MI mostly because patients who simultaneously occlude multiple coronary arteries present with cardiogenic shock or cardiac arrest/sudden death (Pollak & Brady, 2012).

ST depression is less frequent in myocarditis/myopericarditis (with the exception of lead aVR); when present in patients simultaneously displaying ST elevation, it likely represents a reciprocal change and suggests MI (Pollak & Brady, 2012). However, nonspecific ST depression has been reported in FM (Ginsberg & Parrillo, 2013), where it can represent one of the earliest clinical signs (Wang et al., 2019). Moreover, De Winter sign (defined as a 1–3 mm upsloping ST depression in V1‐V6 that continues into tall, positive symmetrical T waves, generally associated with ST elevation of 1–2 mm in aVR lead and mild ST depression in inferior leads), a well‐described ECG pattern that typically suggests acute occlusion of proximal left anterior descending coronary artery, has also been described in ACS‐like myocarditis (de Winter, Verouden, Wellens, & Wilde, 2008; García‐Izquierdo, Parra‐Esteban, Mirelis, & Fernández‐Lozano, 2018).

## 5 T WAVE

**Figure 5:**
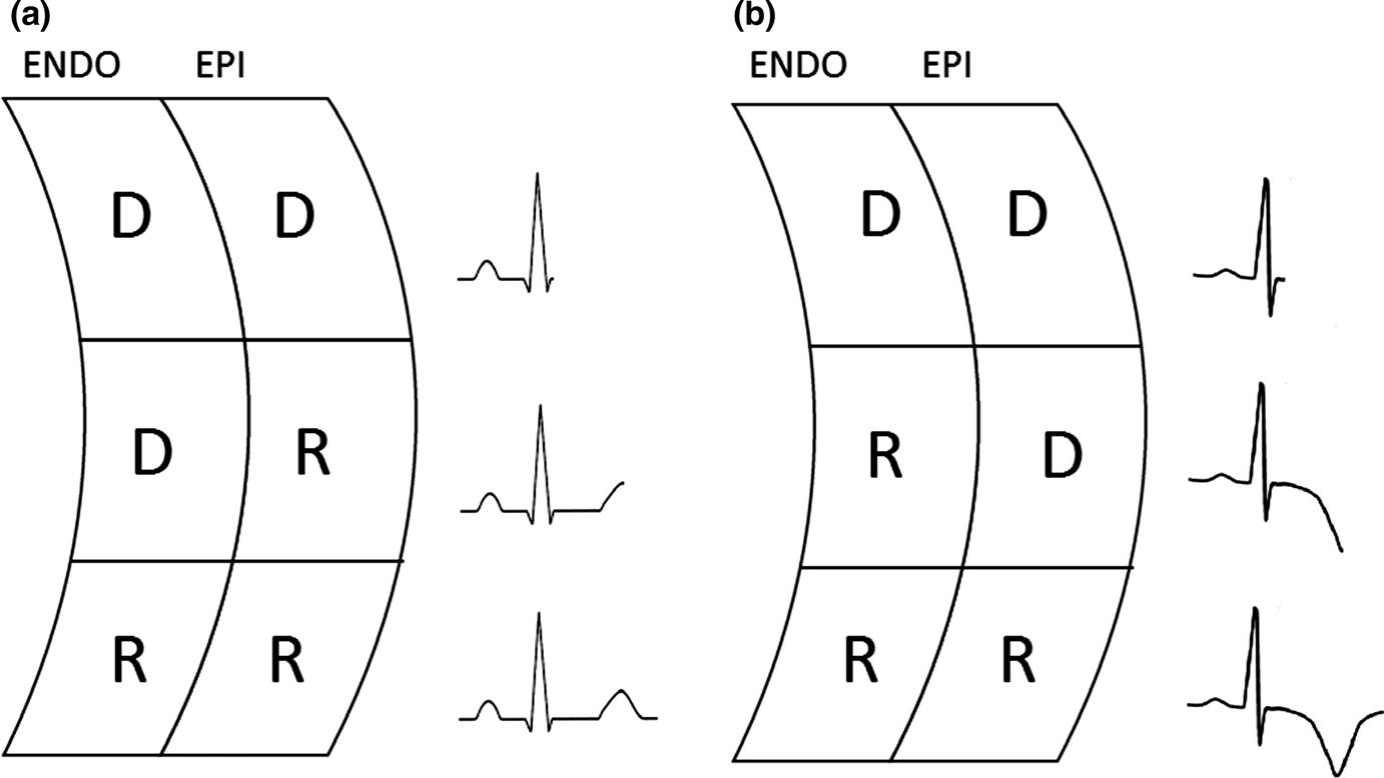
A theory to explain T‐wave inversion in myocarditis. In a normal heart, action potential is longer in endocardial cells as compared with epicardial cells and therefore repolarization begins within the epicardial layer and ends within the endocardial layer, giving rise to a positive T wave (a). In transmural or epicardial myocarditis, myocardial damage causes a prolongation of the action potential within the epicardial layer; consequently, repolarization begins within the endocardial layer and ends within the epicardial layer, giving rise to a negative T wave (b). ENDO = endocardial layer, EPI = epicardial layer, D = depolarized cell, R = repolarized cell

The most common T‐wave change in acute myocarditis is T‐wave inversion. It is one of the possible ECG manifestations of ACS‐like myocarditis, but it can be also present in FM (Chan et al., 1999; Ginsberg & Parrillo, 2013; Punja et al., 2010; Scheffold, Herkommer, Kandolf, & May, 2015). The reported prevalence of T‐wave inversion in patients with acute myocarditis ranges from 9% to 48% (De Lazzari et al., 2016). The possible pathogenic link between myocardial edema/inflammation and repolarization abnormalities remains to be elucidated. One can speculate that the acute inflammatory process causes myocyte repolarization inhomogeneity, either regional or more likely transmural, which gives rise to inversion of T‐wave polarity (see Figure 5) (De Lazzari et al., 2016). As for myocardial ischemia or acute pericarditis, also in acute myocarditis a symmetrical T‐wave inversion (mostly in leads with former ST elevation) seems to be a late ECG alteration (see Figures 1 and 2) (de Bliek, 2018; Nakashima et al., 1994). A study by Di Bella et al. (2012) showed that an inverted T wave was present in 9% of patients at admission but in 57% of patients 48 hr later. It is important noting that a T‐wave inversion in V1 to V3 leads in patients of less than 16 years and a ST elevation followed by T‐wave inversion from V1 to V4 leads in black athletes are acknowledged features of athlete's heart rather than of myocarditis (Eichhorn et al., 2019).

The presence of T‐wave inversion was found to be significantly and independently related to the extent of both myocardial necrosis/scar as assessed by MRI in LGE sequences and myocardial edema as assessed by T2‐weighted MRI sequences (De Lazzari et al., 2016; Nucifora et al., 2013). Findings regarding topographic concordance between T‐wave inversion and myocardial damage are less univocal, especially when evaluated with MRI in LGE sequences (De Lazzari et al., 2016); however, there seems to be strong concordance between T‐wave inversion location and distribution of transmural myocardial edema as assessed by MRI in T2‐weighted sequences (De Lazzari et al., 2016). Myocardial edema is, by definition, transient in acute myocarditis and not necessarily associated with irreversible structural myocardial damage (De Lazzari et al., 2016). This could, at least in part, explain why the presence of T‐wave inversion does not provide prognostic information (De Lazzari et al., 2016). On the contrary, a wide QRS‐T angle (≥100°) has been shown to be a significant independent predictor death and HF in a population of patients with acute myocarditis (Chen et al., 2018).

## 6 QT INTERVAL

Another possible ECG alteration in acute myocarditis is QT interval prolongation (Demangone, 2006; Scheffold et al., 2015). Its prevalence is variable (Di Bella et al., 2012; Ramamurthy et al., 1993). Identification of a prolonged QT interval is very important because it represents a well‐known potential arrhythmogenic trigger (Buttà et al., 2014). Accordingly, corrected QT interval prolongation is associated with poor clinical outcome in myocarditis too (Kindermann et al., 2012).

## 7 ARRHYTHMIAS

Acute myocarditis can induce both brady‐ and tachyarrhythmias (Caforio et al., 2013). More common bradyarrhythmias described in myocarditis are sinus arrest, sinoatrial blocks, and atrioventricular (AV) blocks (Caforio et al., 2013; Scheffold et al., 2015). Atrial standstill may be transient or permanent, requiring definitive pacemaker implantation (Larsen et al., 2013; Prabhu, Srinivas Prasad, Thajudeen, & Namboodiri, 2016). First grade, second grade, advanced, or complete AV block are all possible ECG alterations in acute myocarditis. A study by Morgera T et al in a group of patients with active myocarditis showed a prevalence of first grade AV block of 4.5% and a prevalence of advanced or complete AV block of 15.5% (Morgera et al., 1992). AV blocks are very common in Lyme carditis, cardiac sarcoidosis, GCM, and FM (Sawamura et al., 2018; Scheffold et al., 2015). However, also Chagas disease patients display a high prevalence of first degree AV block (Rojas et al., 2018). Prevalence of first degree and complete AV block in Lyme carditis ranges, respectively, between 87% and 90% and between 44% and 53% (Scheffold et al., 2015). All degrees of AV block have been described in patients with cardiac sarcoidosis (Birnie et al., 2014). Reported prevalence of complete AV block is 23%–30%. It often occurs at younger age as compared with complete AV blocks of other etiology. Along with ventricular tachyarrhythmias, complete AV block accounts for 25%–65% of all deaths caused by cardiac sarcoidosis (Sekhri, Sanal, Delorenzo, Aronow, & Maguire, 2011). In GCM, distal AV blocks are described in up to 30% of patients and could represent the only initial manifestation of the disease (Kandolin et al., 2013). Finally, a study by Sawamura et al in patients with FM supported by percutaneous venoarterial extracorporeal membrane oxygenation showed a complete AV block in 40% of all patients and an even higher prevalence in nonsurvivors (Sawamura et al., 2018). On the contrary, high‐degree AV blocks are relatively rare in EM and have been mostly described in cases of acute necrotizing EM (JCS Joint Working Group, 2011). Of note, high‐degree AV blocks have been related to higher morbidity and mortality in all patients with myocarditis (Ogunbayo et al., 2019).

Both supraventricular and ventricular arrhythmias can occur in patients with inflammatory heart disease (Kindermann et al., 2012). The most common ECG abnormality in myocarditis is sinus tachycardia associated with nonspecific ST/T‐wave changes (Punja et al., 2010). Sinus tachycardia mainly reflects the degree of systemic inflammation and/or of hemodynamic impairment and is, therefore, common in FM (Ginsberg & Parrillo, 2013). Beyond sinus tachycardia, other supraventricular tachycardias are described in acute myocarditis, including atrial fibrillation (AF) and atrial flutter (Chan et al., 1999; Spodick, 1998). These arrhythmias are usually found in patients with more severe clinical courses or with underlying cardiac disease and/or in specific conditions such as Chagas disease (Rojas et al., 2018). Their occurrence in acute myocarditis seems to be independent from direct involvement of sinus node by myocardial inflammation (Chan et al., 1999); indeed, they typically develop in patients with underlying structural cardiac disease and/or hemodynamic impairment (Deluigi et al., 2013; Spodick, 1998). Interestingly, AF is an almost universal finding in GCM with isolated atrial involvement due to massive atrial dilatation, atrial wall thickening, and edema (Larsen et al., 2013).

Patients with myocarditis and ventricular arrhythmias may present with a wide spectrum of symptoms ranging from palpitation to syncope. Ventricular arrhythmias are frequent in FM and, particularly, in infiltrative myocarditis, where they can occur even in patients with normal ventricular function (Granér et al., 2007). Prevalence of ventricular arrhythmias during the index hospitalization in GCM ranges between 14% and 22%, and the risk of life‐threatening ventricular arrhythmias exceeds 50% at 5 years (Cooper, Berry, & Shabetai, 1997; Ekström et al., 2016). In EM, 11% of patients developed ventricular arrhythmias during index hospitalization, with a higher rate when secondary to hypersensitivity reaction (Brambatti et al., 2017). Due to the high prevalence of ventricular arrhythmias in infiltrative myocarditis, they represent an indication for endomyocardial biopsy in patients with acute myocarditis, as for high‐degree AV blocks (Magnani & Dec, 2006).

Ventricular arrhythmias are an important cause of sudden cardiac death in myocarditis, particularly in the presence of large areas of LGE at cardiac MRI (Neilan et al., 2015). When refractory to defibrillation, ventricular tachyarrhythmias are associated with poor short‐term prognosis and percutaneous cardiopulmonary support should be considered (Priori et al., 2015). Ventricular arrhythmias at presentation predict the occurrence of sudden cardiac death and ventricular tachycardia during long‐term follow‐up in GCM (Ekström et al., 2016); as a consequence, the presence of malignant ventricular arrhythmia might warrant earlier consideration of an implantable cardioverter‐defibrillator in GCM (Priori et al., 2015). On the contrary, acute‐phase ventricular arrhythmias in viral myocarditis tend to be self‐limiting and, if promptly managed, do not bear significant long‐term prognostic value (Peretto et al., 2019). Therefore, implantable cardioverter‐defibrillator should be deferred until resolution of the acute episode; bridging the critical period to full recovery by a wearable cardioverter‐defibrillator vest appears to be a promising therapeutic option in this setting (Priori et al., 2015).

## 8 CONCLUSIONS

Acute myocarditis represents a challenging diagnosis as there is no pathognomonic clinical presentation and the disease may mimic a variety of noninflammatory myocardial diseases, including ACS. ECG in patients with myocarditis can display a variety of abnormalities and, therefore, represents an useful screening tool. Moreover, some ECG abnormalities are independent predictors of adverse prognosis in patients with acute myocarditis (see Table 1). These data could be very useful in clinical practice for a patient‐tailored approach in order to decide appropriate therapy, length of hospitalization, and frequency of follow‐up.

**Table 1:** Synopsis of typical ECG features useful for differential diagnosis with MI and of prognostic role of ECG in acute myocarditis

	Typical features useful for differential diagnosis with MI	Worse prognosis	Better prognosis
PR segment	Depression in both precordial and limb leads	/	/
	Depression in leads with ST segment elevation		
	Elevation in aVR lead		
QRS complex	/	Q wave: especially when associated with ST segment elevation	/
	/	Wide QRS complex	/
ST segment elevation	Pericarditis pattern (see text for details)	/	Pericarditis pattern with terminal QRS notching or slurring
T wave	/	QRS/T angle ≥ 100°	/
QT interval	/	Prolonged QT interval	/
Arrhythmias	/	High‐degree AV blocks	/
		Malignant ventricular tachyarrhythmias	

Abbreviations: AV, atrioventricular; ECG, electrocardiogram; MI, myocardial infarction.

## CONFLICT OF INTEREST

None.
